# Design of a multitissue osteoarticular 3D-Printed knee model for initial training in meniscal repair

**DOI:** 10.1051/sicotj/2026046

**Published:** 2026-09-18

**Authors:** Manon Pignero, Javier Arduengo Garcia, Jean Yves Hascoet, Cyrille Decante, Julien Berhouet, Herve Thomazeau, Vincent Biscaccianti, Vincent Crenn

**Affiliations:** 1 Nantes Université, CHU Nantes, Department of Orthopaedic F-44000 Nantes France; 2 Ecole Centrale de Nantes F-44000 Nantes France; 3 Nantes Université, CHU Nantes, Department of Pediatric Orthopaedic Surgery F-44000 Nantes France; 4 Centre Hospitalier Regional Universitaire de Tours, Department of Orthopaedic 37000 Tours France; 5 Centre Hospitalier Universitaire de Rennes, Department of Orthopaedic 35033 Rennes France

**Keywords:** Simulation, Arthroscopy, Meniscal repair, 3D printing, Knee

## Abstract

*Purpose*: Meniscal preservation is essential, given the degenerative risk associated with meniscectomy. Consequently, meniscal repair is a key technical skill to be acquired early during surgical training. However, resident education is limited by restricted access to human models. While artificial simulators represent an alternative, their use is often constrained by high cost and limited biomechanical or arthroscopic realism. The aim of this project was to design and evaluate a 3D-printed arthroscopic simulator dedicated to meniscal repair, developed through a collaboration between orthopedic surgeons and engineers, in order to provide an accessible and realistic educational tool. *Methods*: Modeling and design were jointly performed by orthopedic surgeons and engineers based on segmentation of medical imaging data. A complete lower limb was created, incorporating a removable capsulomeniscal complex. Osseous and cartilaginous structures were produced by direct 3D printing, while ligaments and the capsulomeniscal complex were molded in silicone using 3D-printed templates. This removable complex differentiates peripheral capsular insertions from meniscal roots, reproducing the anatomical constraints. The model was assessed through a subjective evaluation of arthroscopic realism during meniscal suturing procedures by two independent knee surgery experts. *Results*: The prototype reproduces a complete lower limb articulated around a fixed femoral pivot, with near-physiological hip and knee motion. It allows dry arthroscopy for various meniscal tear patterns and requires Cabot and valgus maneuvers for optimal femorotibial exposure. Ramp lesions can be repaired via a postero-medial approach using a hook instrument, whereas osseous procedures such as meniscal root reinsertion remain unfeasible. Both evaluators rated as “very realistic” the anatomy, haptic feedback, and ramp lesion suturing. Overall, arthroscopic realism was rated as “very realistic” by both. *Conclusion*: This study presents a realistic 3D-printed simulator for meniscal repair, addressing a clear educational need. Further development and large-scale validation studies involving trainees are required to confirm its pedagogical relevance. *Study type / Level of evidence*: Experimental design study / Level IV.

## Introduction

The “save the meniscus” doctrine [1] is now central to the management of meniscal lesions. Supported by the literature and by scientific societies such as ESSKA [2], this conservative approach prioritizes meniscal repair in order to preserve the biomechanical function of the meniscus and prevent post-meniscectomy degeneration [3]. This imperative for preservation strengthens the need for safe, simulation-based surgical training, which has become a cornerstone of surgical education [4]. While cadavers historically formed the basis of training, stricter regulations and international variability have limited their use, leading to the expansion of artificial simulators. Low-fidelity simulators, which are inexpensive and effective for learning basic techniques, remain far from actual surgical conditions [5, 6], while high-fidelity simulators [7], often very costly and incorporating virtual reality, are inaccessible to many training centers.

In orthopedics, arthroscopy training is mandatory, yet most senior residents do not feel able to practice independently without supervision [8]. Virtual-reality simulation tools may improve basic skills such as triangulation [9], but remain insufficient for complex procedures involving implantable devices, notably due to the absence of haptic feedback. Non-organic 3D training models constitute a durable solution but face limitations: lack of biomechanical fidelity, high cost, absence of customization, and a limited industrial supply. Existing three-dimensional (3D) printed models are often rudimentary or insufficiently realistic, particularly in arthroscopy [10–12].

Meniscal lesions vary in location, orientation, and mechanism, and meniscal suture techniques differ accordingly. Current industrial simulators allow neither training in the repair of ramp lesions nor a realistic representation of the menisco-capsular insertions, which are essential to biomechanical realism. Their absence alters the perceived resistance required when using suture devices and leads to frequent meniscal tearing during training. The lack of complete lower-limb representation limits the reproduction of operative conditions, in which positioning and ligamentous constraints influence instrument handling. Exposure of the medial compartment remains particularly challenging, contributing to diagnostic errors and potential iatrogenic lesions [13]. Finally, their high cost and dependence on manufacturers for consumables restrict their availability in training programs.

The aim of this work was to design and evaluate, through collaboration between surgeons and engineers, a 3D-printed knee arthroscopy simulator specifically dedicated to training in meniscal repair. The hypothesis was to provide a realistic, reproducible, and cost-effective alternative to organic models and virtual simulators.

## Methods

### Study design

This was a three-year prospective research-and-development study conducted through a collaboration between a university hospital orthopedic surgery team and an engineering team from an engineering school. All experimental devices were entirely designed and manufactured in-house, with no commercial use and no financial benefit for the authors. The project was funded through a regional call for proposals in educational research, with co-funding from a healthcare simulation center and resources from a mechanical engineering laboratory. All patient-related data were collected in accordance with the ethical principles of the Declaration of Helsinki, after obtaining informed consent explicitly authorizing their use for scientific research and dissemination in publications.

### Initial concept

The specifications aimed to realistically reproduce the full meniscal repair procedure, incorporating the exposure constraints of the joint compartments and the manipulation of the lower limb. The model had to represent a full-scale, articulated lower limb with accurate biomechanical properties and physiological ranges of motion at the hip and knee. The femoral pivot was fixed to the support, while the distal extremity remained free to allow mobilization by trainees. The model, therefore, needed to accommodate a removable joint capsule, enabling the use of a new meniscal structure for each training session.

### Modeling

Model development was based on multimodal DICOM imaging (radiographs, CT scan, and MRI) obtained from a single, trauma-free 25-year-old patient. Osseous structures were segmented using a semi-automated method, whereas fine or low-contrast anatomical structures required a hybrid approach combining manual landmark placement and CAD-based surface reconstruction. Model segmentation and design were performed collaboratively by engineers and orthopedic surgeons using CAD/CAM software.

Meniscal modeling relied on manual landmark-based reconstruction due to the limitations of automated segmentation, allowing accurate reproduction of meniscal curvature and concavity. Meniscal roots were modeled separately to account for their specific osseous insertions. Ligament modeling was simplified to proximal and distal insertions, while patellar and quadriceps tendons were reconstructed in greater detail. Finally, joint capsule modeling required a combined imaging- and anatomy-based approach, integrating MRI data with direct anatomical observations by an expert surgeon.

**Figure 1:**
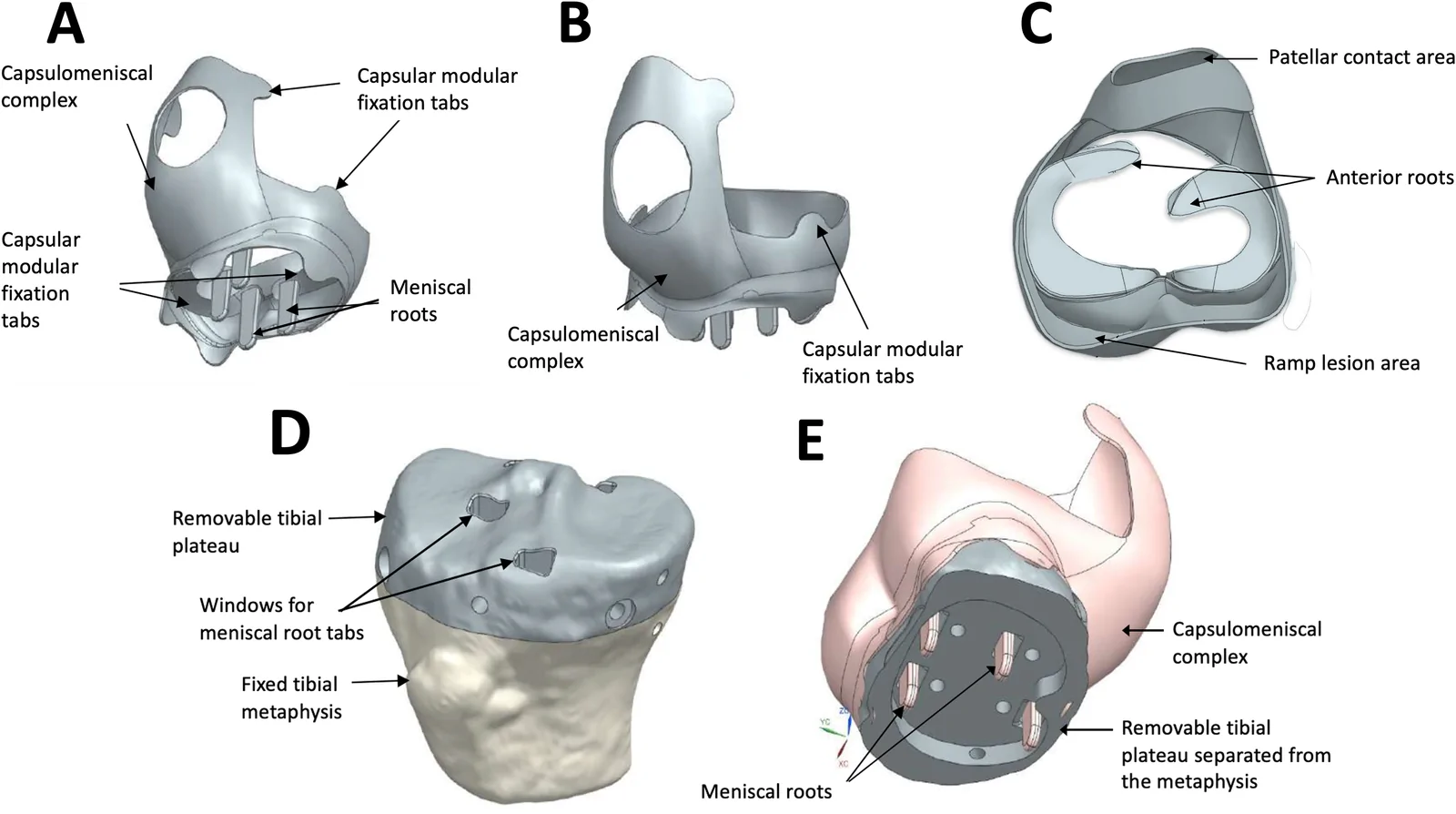
Modeling of the capsulo-meniscal complex and the removable tibial plateau. A: Inferior view of the CMC. B: Three-quarter view of the CMC. C: Superior view of the CMC. D: Three-quarter view showing the removable tibial plateau in grey, fixed onto the tibial metaphysis in beige. E: Tibial plateau detached from its metaphyseal base, attached to the CMC through dedicated windows allowing passage of the meniscal roots. These roots are subsequently anchored by screwing the fixation tabs. The plateau can then be secured to the remaining tibial structure.

The peripheral insertions of the menisci rely largely on the menisco-capsular and coronary ligaments, whereas their central anchorage depends on the meniscal roots. Consequently, it was decided to model a unified capsulo-meniscal complex (CMC) (Figure 1), whose attachment to the articular skeleton is ensured both by fixation of the anterior and posterior meniscal roots and by the capsular insertion on the femoral condyles and the removable tibial plateau (Figure 1).

The mechanical axes of the lower limb were defined in the frontal plane by aligning the bone model with the standing long-leg radiograph. The tibial axis and the frontal anatomical axes of the femur were determined on the prototype. A sagittal projection was then performed to characterize the femoral curvature, while an axial projection was used to assess femoral neck anteversion.

### Design

**Figure 2:**
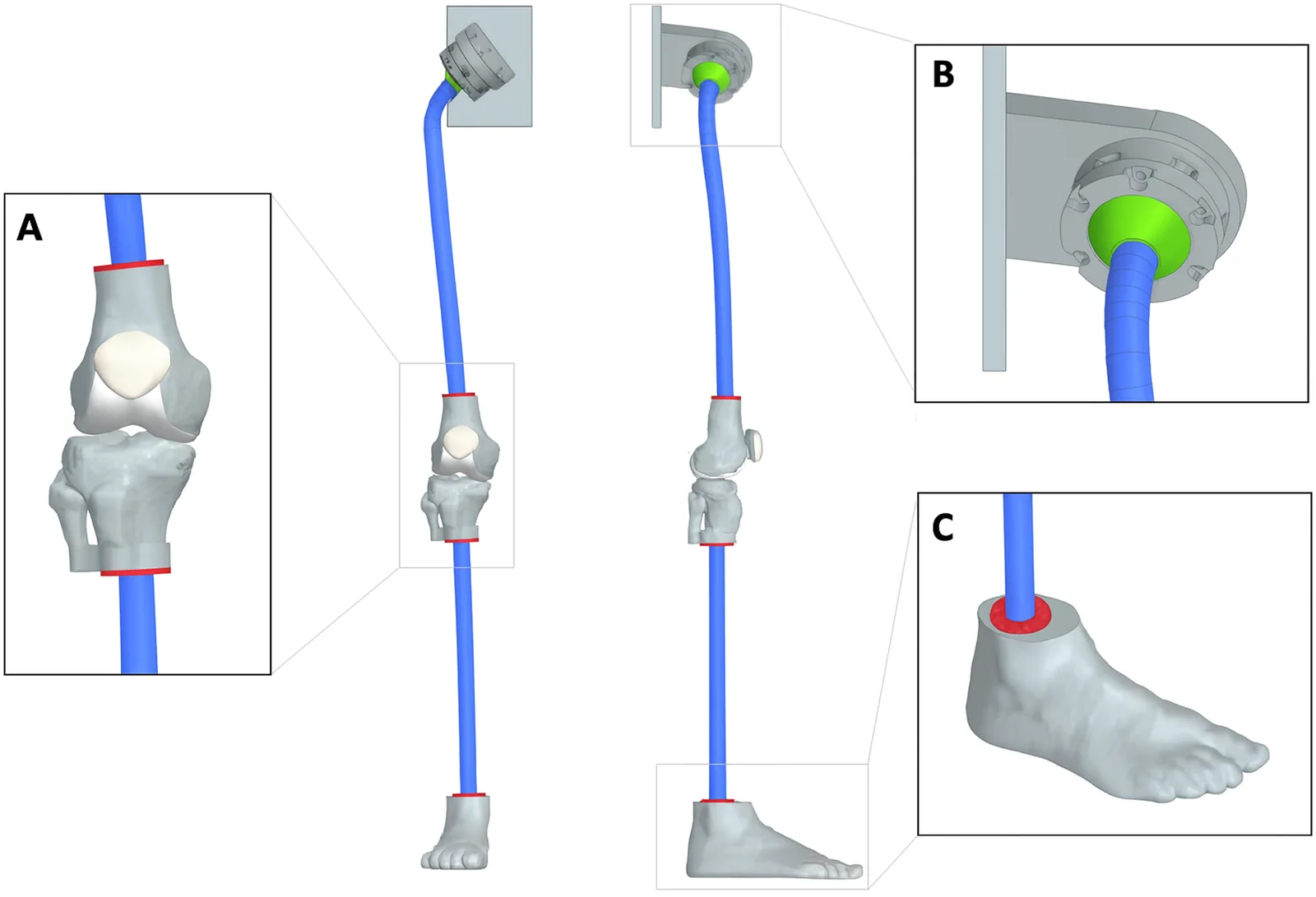
Final 3D modeling of the permanent structure. On the left, frontal view; on the right, lateral view. The femoral and tibial diaphyses are shown in blue. The machined connecting components are shown in red. A: Frontal view of the knee joint modeling. The fixed metaphyseal–epiphyseal structures of the femur, tibia, and fibula are shown in grey. The patella is modeled anterior to the femoral trochlea. Cartilage is represented in white. B: Lateral view of the hip joint modeling, represented by the femoral head in green. C: Three-quarter view of the foot modeling, shown in grey.

The fixed structure (Figure 2) was custom-built to reproduce physiological hip and knee kinematics. The femoral head was machined to allow appropriate hip ranges of motion, while the femoral and tibial shafts were manufactured from stainless-steel tubes. The femoral tube was bent to reproduce femoral neck orientation and diaphyseal curvature, based on population reference values. Components were assembled using custom-machined connectors. The foot and knee epiphyses were produced by FDM 3D printing in PLA, whereas the femoral, tibial, and patellar cartilage surfaces, which required higher surface quality, were manufactured by stereolithography (SLA) using a liquid resin.

The ligamentous structures corresponding to the medial collateral ligament (MCL) and lateral collateral ligament (LCL) were produced using hermetically sealed 3D-printed SLA molds, into which a two-component silicone (ResChimica^®^, Shore A30) was injected. Their fixation in the model was achieved by screwing them into the corresponding anatomical insertion sites [14, 15].

The extensor mechanism was reproduced using an overmolding technique. An SLA-printed patellar component was placed in a 3D mold and overmolded with Shore A10 silicone to recreate the peri-patellar soft tissues and ensure functional continuity. The assembled extensor mechanism was fixed to the model at two anchoring points, proximally on the femoral shaft to reproduce quadriceps traction, and distally on the tibial tuberosity to replicate patellar tendon insertion.

The ACL and PCL were produced by mixing two components following the previously described fabrication method. A review of the literature on the mechanical properties of the central pivot guided the selection of inextensible, traction-resistant materials [16].

The central pivot was modeled as a non-elastic structure under standard clinical conditions by combining a rigid internal core made of metal wires with a flexible Shore A10 silicone coating. To best reproduce ligament tension in the central pivot and mimic the mechanical behavior of the native knee, a fixation technique inspired by ACL and PCL reconstruction procedures was adopted [17, 18]. The femoral and tibial epiphyses were drilled to create tunnels whose intra-articular outlets corresponded to the anatomical insertion points of the ACL and PCL. After demolding, the ligaments were inserted into these tunnels and secured extracortically using a screw-based fixation system. This configuration allows tensioning of the PCL at 90° of flexion and of the ACL at 30° of flexion.

Designing an educational tool for training in meniscal repair requires a faithful reproduction of the biomechanical, haptic, and visual behavior of the meniscus. Physiological properties, particularly elasticity and hardness, were therefore sought. Owing to the lack of precise data in the literature and the absence of an industrial material capable of reproducing meniscal anisotropy, the Young’s modulus was not used as a selection criterion. Material choice was instead based on practical parameters: cost, accessibility, ease of use, surface finish, hardness, and quality of haptic feedback. Two-component ResChimica^®^ silicones (Shore A10, A20, A30) were injected into the capsulo-meniscal mold, integrated into the finalized simulator, and then tested under arthroscopic conditions by two orthopedic surgeons, one senior and one in training. Evaluation focused on haptic feedback and, in particular, the perceived sensation during “all-inside” meniscal suturing using rapid fixation devices with anchors. Following this assessment, ResChimica^®^ Shore A20 silicone was selected for final manufacturing.

The complexity of the anatomical relationships between the menisci and adjacent structures makes a complete representation of all meniscal attachments unattainable. However, the main fixation points include capsular insertion via the menisco-capsular ligament, osseous anchorage through the meniscal roots, and tibial fixation via the menisco-tibial ligament [19]. Based on these considerations, a unified capsulo-meniscal structure was modeled. Given the fineness and smooth texture required, direct 3D printing with standard SLA printers was unsuitable. To ensure reproducibility, a mold was designed using SLA 3D printing in liquid resin, intended for silicone injection to produce the final structure.

After three hours of polymerization, the capsulo-meniscal complex can be removed from the mold. This component is then integrated into the simulator. The tibial plateau is unscrewed, allowing insertion of the four silicone tabs (two anterior and two posterior) corresponding to the meniscal root insertion sites. These tabs are fixed by screws beneath the tibial epiphysis (Figure 1). Once the removable tibial plateau is repositioned on its support, the complex is secured to the external edges of the plateau using a screw fixation system (Figure 1).

### Iterative adjustment process

Initial testing revealed tearing of the silicone capsulo-meniscal component during all-inside suturing. Local reinforcement was achieved by embedding sterile gauze within the silicone mold. To improve visual realism, a white Shore A20 silicone (Prodemmia^®^) replaced the initial blue-tinted material. CAD modifications were required to prevent anterior impingement, leading to a shortened anterior capsule. Persistent hyperextension was corrected by adding a posterior tensioning cord, limiting extension to 0°.

### Arthroscopic assessment

Two independent senior orthopedic surgeons specialized in knee arthroscopy, and not involved in the design of the study, performed a subjective evaluation of the model. Assessments were conducted independently and in a blinded manner between evaluators and consisted of a dry arthroscopic examination using a 30° portable arthroscopy camera system. The evaluators assessed the visual, haptic, and anatomical realism of the model, as well as the intraoperative sensations experienced during simulated meniscal repair procedures, including a vertical suture of the mid-body of the lateral meniscus using an all-inside technique and the repair of a ramp lesion through a posteromedial approach. Following the procedures, the evaluators completed a 10-item questionnaire rated on 5-point Likert scales (from 1 = “not realistic at all” to 5 = “highly realistic, close to real”), allowing assessment of the perceived realism of the model compared with a human knee.

## Results

### Model presentation

**Figure 3:**
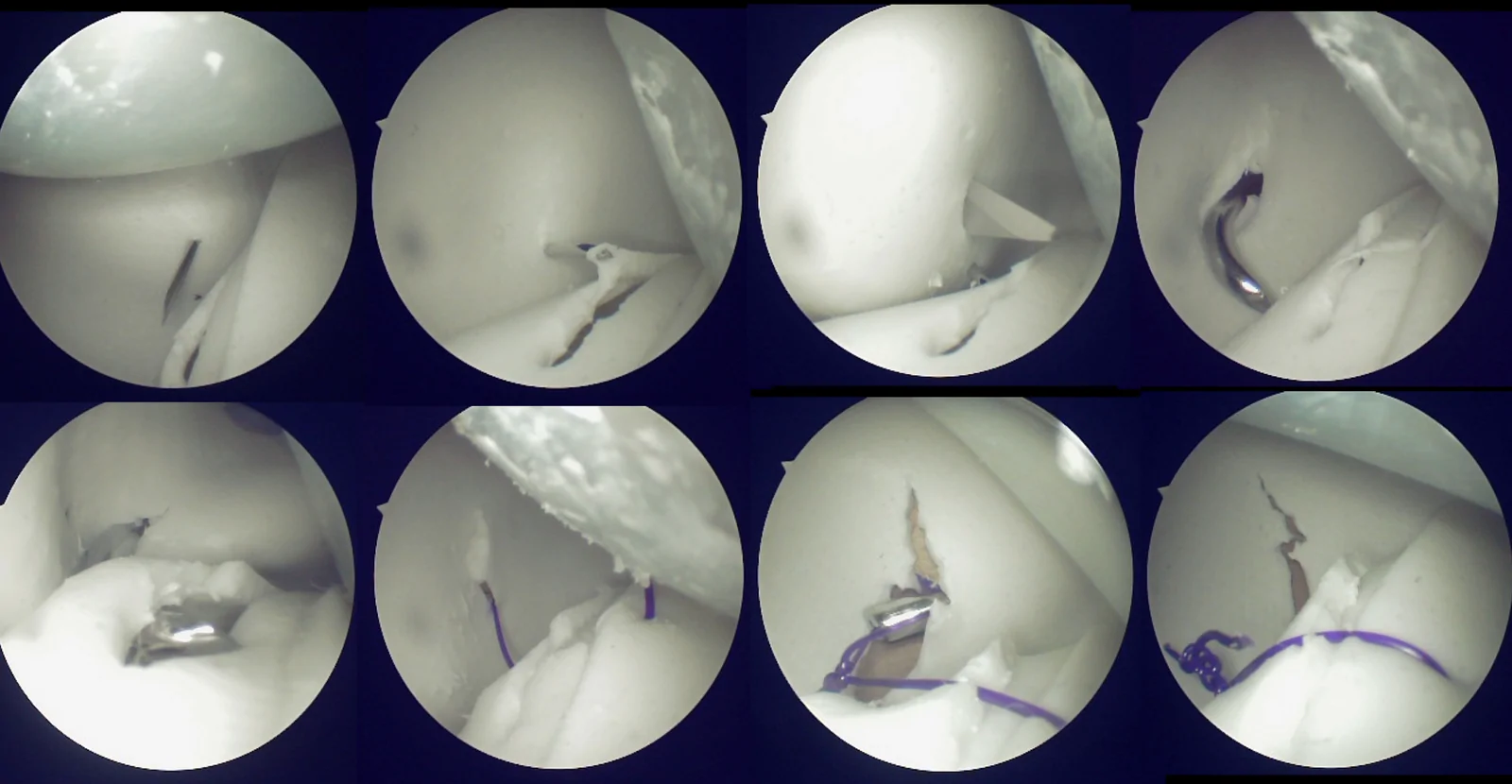
Successive steps of ramp lesion repair. From left to right and top to bottom. 1, Needle triangulation to identify the entry point. 2, Visualization of the lesion to be repaired using the needle. 3, Creation of the posteromedial portal with a scalpel, following the needle trajectory. 4, Insertion of the suture hook through the posteromedial portal. 5, Passage of the hook through the ramp lesion. 6, Withdrawal of the hook, leaving the PDS suture in place on both sides of the lesion. 7, Knot tying and tightening using a knot pusher. 8, Final appearance after lesion repair.

The suture sequences for the ramp lesion on the simulator are shown in Figure 3. They illustrate, in chronological order, the steps of the hook-based repair performed by a knee surgeon.

**Figure 4:**
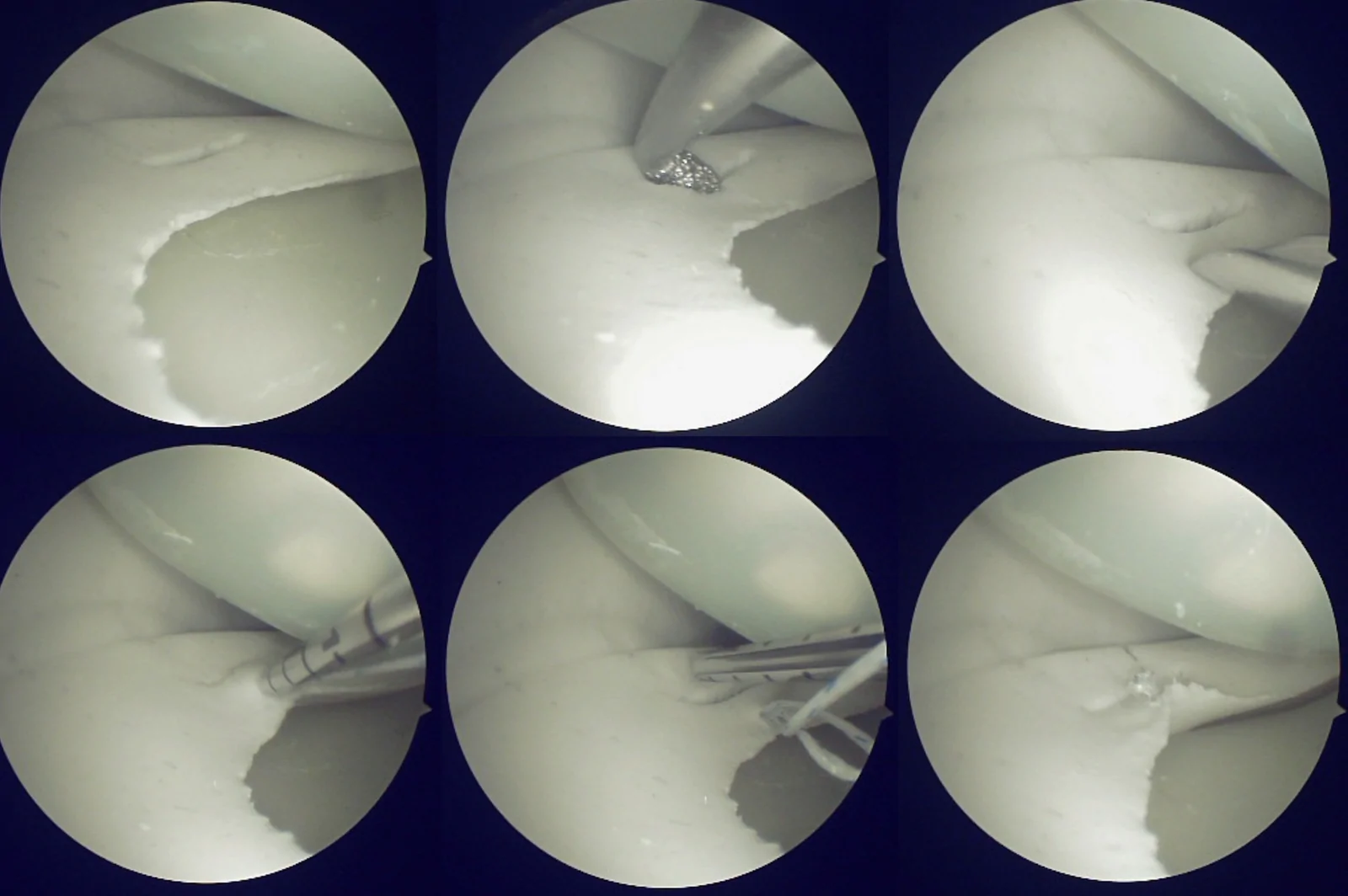
Successive steps of vertical repair of a mid-body lateral meniscus lesion. From left to right and top to bottom. 1, Visualization of the full-thickness vertical lesion. 2, Refreshing of the lesion using a diamond rasp. 3, Insertion of the FastFix^®^ device guiding cannula. 4, First FastFix^®^ passage beneath the lesion. 5, Second FastFix® passage above the lesion. 6, Final appearance of the repair after knot tightening and suture trimming.

The sequences for the vertical meniscal repair are shown in Figure 4.

### Assessment results

Both surgeons rated the external appearance and intra-articular arthroscopic anatomy of the model as realistic. Meniscal roots and body representation, as well as haptic feedback during meniscal probing and traction, were judged highly realistic by both evaluators. Ligament tension during probing was rated from realistic to highly realistic. Technical procedures, including all-inside meniscal repair and ramp lesion suturing, were considered realistic to highly realistic, as were dynamic maneuvers. Overall, the arthroscopic procedure was rated as highly realistic. One evaluator noted excellent haptic feedback, with minor limitations in frontal plane laxity, deemed acceptable for a training model (Table 1).

**Table 1:** Arthroscopic assessment of simulator realism performed by two senior expert knee surgeons. Each item was scored on a 5-point Likert scale ranging from 1 (“not realistic at all”) to 5 (“highly realistic”).

Test (Likert scale, 1 to 5)	Surgeon 2	Surgeon 1
Meniscal palpation and traction sensation	5	5
ACL/PCL tension assessed with the probe	5	5
All-inside meniscal repair	5	5
Ramp lesion repair	4	5
Realism of the meniscal roots	5	5
Realism of the meniscal segments	4	5
Realism of dynamic maneuvers (Cabot/valgus)	4	4
External appearance	4	4
Intra-articular appearance	4	4
Overall impression of arthroscopic realism	5	5

## Discussion

The objective of this study was to design, through 3D printing, a simulator dedicated to introducing residents to meniscal repair. Although scientific societies recommend meniscal repair rather than meniscectomy because of its arthrogenic consequences, it is well established that arthroscopy can lead to iatrogenic chondral lesions [20] with a risk that is even higher when the operator is young and inexperienced [21]. It therefore appears essential for each university center to be equipped with accessible, low-cost, and anatomically faithful simulators, enabling mastery of the procedure prior to any intervention on a patient.

### Simulator limitations

The simulator allows training in meniscal repair for most lesions not requiring bony procedures, including radial, vertical, bucket-handle, flap, and ramp tears. However, it does not reproduce meniscal root reinsertion or ligament reconstruction. Although integration of artificial bone substitutes could broaden their indications, such solutions require advanced 3D-printing expertise that is generally inaccessible to trainees. In addition, the use of the simulator requires a dry arthroscopy camera system, with costs ranging from 100 to 2000 euros.

Intra-articular exposure is facilitated by the absence of the Hoffa fat pad, an anatomical structure that frequently limits visualization during early arthroscopic procedures. While this simplifies exposure, it reduces anatomical realism and removes a key constraint normally encountered in clinical practice. Although the final cost of the simulator is lower than most commercial solutions, its development required substantial initial financial and technical investment.

Material selection for the menisci and ligaments was based on empirical haptic assessment by expert surgeons due to the lack of standardized biomechanical data. Some knee models described in the literature have adopted a similar approach. For example, Ruiz and Dhaher [22] developed a 3D-printed anatomical knee model based on MRI data. For the menisci, they selected a material with Shore 28A hardness, which is close to the Shore 20A materials used in the present work.

The collateral ligaments were produced in silicone to ensure joint stability while preserving arthroscopic exposure, with their tension empirically adjusted to achieve an optimal balance between mechanical realism and accessibility. Future developments may integrate disposable fibrillar ligaments to enable training in the pie-crusting technique.

Early testing revealed insufficient fixation of all-inside anchors within the capsulo-meniscal complex, necessitating reinforcement with a fabric mesh and increasing fabrication complexity.

Due to time constraints, the evaluation was limited to a preliminary and subjective assessment performed by two senior expert surgeons. Consequently, the conclusions drawn from these findings should be interpreted with caution. Furthermore, as the simulator has not yet been evaluated by surgical trainees, no conclusions regarding its pedagogical validity or educational effectiveness can currently be established. Additional studies involving residents and objective performance outcomes are therefore required.

### Strengths

This knee model was designed to be accessible and reproducible. It relies on silicone injection into 3D-printed molds, allowing the fabrication of modular structures (capsule, ligaments, menisci) without requiring a 3D printer for the end user. The initial production cost of the simulator was approximately €1300. However, once the molds have been manufactured, the self-production cost of a replaceable articular capsule is less than €10 per unit. Cost-effective and easy to reproduce, it enables trainees to replace interchangeable components without relying on specialized manufacturers.

In the literature, low-cost and easy-to-use arthroscopy simulators are generally associated with limited fidelity. For example, the 3D-printed model proposed by Ferràs-Tarragó et al. [11] enables inexpensive introductory triangulation training but remains highly simplified, with no soft tissues or anatomical structures. In contrast, the knee model presented in this study aims to combine accessibility with greater anatomical and biomechanical realism, providing a training experience closer to real clinical conditions.

From an anatomical standpoint, this device accurately reproduces meniscal insertions, including the ramp region, which is typically absent from commercially available simulators. It is also, to our knowledge, the only 3D-printed model integrating menisco-capsular attachments, whereas most simulators restrict fixation to the meniscal roots; for example, the multilayered static 3D-printed model developed by Ruiz and Dhaher [22], does not reproduce either the ramp region or the menisco-capsular junction.

Among recent 3D-printed arthroscopy simulators, none reproduces the entire limb, despite the importance of limb positioning and manipulation in determining arthroscopic access. Cai et al. [12] developed a realistic and low-cost hip simulator combining bone and soft tissues with modular components, but the simulation remains static, without dynamic constraints.

Similarly, Biggs et al. [10] proposed a 3D-printed shoulder arthroscopy simulator that is simple, modular, and affordable, but limited by the absence of dynamic soft-tissue behavior and by its restriction to a single procedure, in contrast with the broader educational scope of the knee model presented in this study.

Among the potential avenues for development, adaptation of the molds could enable production of discoid menisci, allowing training in saucerization, a relatively rare procedure currently absent from existing simulators. These menisci are also frequently associated with menisco-capsular lesions requiring specific repair [23]. In the presented model, the structural linkage between meniscus and capsule would facilitate the reproduction of such lesions and provide a valuable educational tool for learning procedures that are infrequent but essential for future knee surgeons.

Table 1 shows that the simulator was rated as faithful by experts, particularly for meniscal suturing and overall arthroscopic realism. Visual aspects and dynamic maneuvers received slightly lower scores, indicating potential areas for improvement in visual rendering and joint mobility reproduction.

Finally, the arthroscopic evaluation of the simulator should be complemented by objective metrics (operative time, technical success rate, improvement after training) in order to confirm its educational validity. Comparative studies, particularly on cadaveric specimens, comparing trainees trained on the simulator with a control group, would help determine the device’s impact on technical performance and procedure time, as previously demonstrated in other simulation settings [24].

## Conclusion

This study led to the development of the first realistic meniscal repair training model produced by 3D printing, designed to address an identified need in surgical education and in the preservation of meniscal and cartilaginous tissue. However, the present evaluation remains preliminary, relying on a limited subjective assessment by two senior expert surgeons, and therefore, the findings should be interpreted with caution. Although this simulator is promising and represents a significant advance as the first device specifically dedicated to teaching meniscal repair using 3D printing, it remains improvable. Future developments should aim to expand its capabilities, particularly by incorporating bony procedures that are currently not feasible. Additional studies are required to rigorously assess its educational validity and its impact on the acquisition of technical skills in meniscal arthroscopy.

## Data Availability

Data are available on request from the first author.
